# From experience to expectation: The reverse effect of power on purchasing impulsiveness

**DOI:** 10.3389/fpsyg.2023.1094536

**Published:** 2023-03-08

**Authors:** Yanzhi Wang, Tang Yao, Qi Qiu

**Affiliations:** ^1^School of Management, Tianjin University of Commerce, Tianjin, China; ^2^School of Economics and Management, Beihang University, Beijing, China; ^3^College of Business Administration, Capital University of Economics and Business, Beijing, China

**Keywords:** impulsive purchase, power, power expectation, deservingness, normative assessment

## Abstract

**Introduction:**

Previous literatures have mainly explored the impact of the experience of power on impulsive buying, but have ignored the impacts of the expectations of power. The purpose of this research is to delineates a two-facet portrait of power in the role of affecting purchase impulsiveness by proposing a theoretical extension from the experience of power to the expectations of power.

**Methods:**

Four laboratory experiments were developed that used ANOVA to verify the hypothesis. A moderated mediation path model was established including the experience of power, product attribute, the expectations of power, deservingness, and purchasing impulsiveness as observed variables.

**Results:**

The results revealed that powerless consumers are more likely to impulsively buy hedonic products; while powerful consumers prefer to impulsively buy utilitarian products. However, when focusing on the expectations of power, powerless consumers feel a lower perception of deservingness, which reduces their impulse to buy hedonic products. In contrast, when powerful consumers imagine how powerful people should behave in consumption, they will experience a higher sense of deservingness and increase purchasing impulsiveness for hedonic products. The underlying mechanism is that deservingness plays a mediation role in the three-way interaction impacts of the experience of power, product attribute, and the expectations of power on purchasing impulsiveness.

**Conclusion:**

The current research formulates a new theoretical perspective on the relationship between power and purchasing impulsiveness. An experience-expectation model of power is presented that proposes consumers’ purchasing impulsiveness can be affected both by the experience of power and the expectations of power.

## Introduction

Impulse buying is a pervasive and distinctive aspect of consumers’ lifestyles and also a focal point of considerable marketing management activity (Rook, 1987). The data show that it accounts for as much as 62% of traditional supermarket sales and 80% of all sales in certain product categories (Luo, 2005), within the huge e-commerce market, consumers often make spontaneous, unplanned, unreflective, and unthoughtful impulse purchases (Habib and Qayyum, 2018). Reports from Internet Retailer (2019) indicated that Alibaba and Amazon jointly created a huge sales volume of $1.13 billion in 2018 (Li et al., 2021), of which impulse purchases contributed a large portion of sales. Scholars from different disciplinary backgrounds have explored the drivers of impulsive buying (Luo, 2005; Amos et al., 2014; Chen and Ku, 2021; Li et al., 2021; Chen et al., 2022), especially the impacts of some psychological characteristic such as ability to regulate emotion (Li et al., 2017), anticipated regret (Li et al., 2021), and susceptibility to influence (Luo, 2005).

As a key psychological factor, it seems that the power takes a leading position in impulsive buying behavior. The notion of power is a critical dimension of the judgments or decisions to be made (Li et al., 2022), having or lacking power has transformative effects on consumers’ information processing and decision-making (Keltner et al., 2003; Rucker and Galinsky, 2008; Rucker et al., 2012; Garbinsky et al., 2014). Scholars consistently document the importance of the experience of power in influencing impulsive buying (e.g., Keltner et al., 2003; Smith and Trope, 2006; Jin and Zhu, 2016), but the studies have not achieved a convergent conclusion. Construal level theory, for example, suggests that power increases psychological distances which enable powerful people to make decisions at high construal level, as a consequence, powerful people are not easy to buy impulsively (Smith and Trope, 2006). Empirical researches on self-control (Jia et al., 2020) and saving behavior (Garbinsky et al., 2014) can provide supports. A second line of research based on the power-approach theory (Keltner et al., 2003) suggests that power activates a general tendency to approach whereas powerlessness activates a general tendency to inhibit. As a result, people having power are more likely to purchase products impulsively, while those lacking power are less likely to be impulsive in consumption behavior (Galinsky et al., 2003). A third effort to understand the experience of power has examined the link between power and the types of products purchased on impulse. Jin and Zhu (2016) proposed that powerful individuals are more likely to buy utilitarian products on impulse, while powerless individuals are more likely to buy hedonic products on impulse. The mechanism is that the fluency of information processing leads consumers to consider that their impulse purchase decisions are correct. This mechanism is not exactly the same as the impulsive purchase phenomenon in which consumers encounter the internal psychological conflict of whether or not to buy. Although impulsive buying often occurs spontaneously, it is not a completely uncontrollable behavior, but the result of the failure of self-control caused by desire over willpower (Hoch and Loewenstein, 1991; Baumeister, 2002).

In sum, previous power literature commonly explores the impact of the experience of power on impulsive buying. However, power is accompanied by both an experience (the internal psychological and physiological tendencies that activate when one has or lacks power) and expectations (schemas and scripts that related to how people in a given position of power behave) (Rucker et al., 2014). The expectations of power are related to social stereotype of power. In fact, individuals often observe how people with or without power should behave in society, and hold a series of schemas and scripts related to power. Under Chinese culture, high power individuals are perceived to be more capable (Wang et al., 2017), power stereotypes play an important role in consumers’ decision-making (Zhang et al., 2015; Wang et al., 2017; Li et al., 2022). A focus on the expectations of power might yield distinct and novel effects on consumer decision-making (Rucker et al., 2014). Despite the importance of this variable, to our knowledge, scholars have rarely explored whether the expectations of power will have a different effect on the purchase impulsiveness compared to the experience of power.

In this research, we build on previous research on the expectations of power (Rucker et al., 2014; Wang et al., 2017) to suggest that reminders of the expectations of power promote a normative evaluation of purchase impulsiveness, which guides consumers’ decision making toward impulsive buying (Rook and Fisher, 1995; Kivetz and Zheng, 2017). Theoretically, we propose an associative mechanism to suggest that high power is associated with greater “I deserve” in the face of hedonic product temptations. This associative account, as we describe subsequently, is consistent with the idea that activating the expectations of power triggers the justification of impulsive buying. When normative evaluation of impulse buying is activated through salient low deservingness, powerless individual is less likely to engage in hedonic impulsive buying. Consequently, focused on the social stereotype of powerless or powerful people, people with low power tend to reduce the impulse desire to buy hedonic product, on the contrary, people with high power will increase their impulsive desire to buy hedonic products.

Our findings add to the literature in several important ways, we are the first to examine the impact of the expectations of power on the impulse to buy hedonic products. Meanwhile, this research proposes a theoretical research framework including the dual perspectives of the experience of power and the expectations of power, which can integrate the existing contradictory research results on the relationship between power and impulse purchase. In addition, we identify the sense of deservingness as a heretofore unexamined process underlying the influence of engaging in the expectations of power on purchase impulsiveness. Taken together, our results suggest that consumers derive the justification of impulse buying of hedonic products when they shift the focus from the experience of power (how I feel) to the expectations of power (how I should behave), their impulse willingness to buy hedonic products is related to the results of normative evaluation.

## Literature review and research hypotheses

### The experience of power versus the expectations of power

Power refers to the capacity to influence other people, it emerges from control over valuable resources and the ability to administer rewards and punishments (Keltner et al., 2003). Power is often conceived of as a structural variable and as a property of social relationships, can also become a psychological property of the individual (Galinsky et al., 2003). First, power refers to the ability of an individual to be independent or not be affected by others. Second, power is related to long-term social status, economic status, and perception of controlling over others related to one’s position in an organization. “By creating a rank-ordering collection of individuals, power serves as a social tool to organize and structure individuals and groups” (Rucker et al., 2014). Besides, in the temporary perspective, power is a psychological variable which means that people can feel powerless or powerful independent of their structural position (Rucker et al., 2014). For example, recalling a previous episode in which people felt powerless or powerful alters their sense of power. Therefore, the sense of powerless or powerful could be primed by context, role or memory of experienced state of powerless or powerful (Magee and Galinsky, 2008).

A great deal of research has argued that possessing power could produce a variety of effects ranging from perception of price unfairness (Jin et al., 2014), consumer’s feeling of controlling over inanimate objects (Kim and McGill, 2011), preference for small or large objects (Dubois et al., 2012), and consumer’s information processing and status seeking behavior (Rucker et al., 2014). Compared with powerful consumers, consumers who are lack of power may have less sense of control and have negative emotional experiences (Berdahl and Martorana, 2006). Therefore, in order to restore the control and get rid of negative experiences, the compensatory consumer behavior is produced, especially for buying high social-status products (Rucker and Galinsky, 2008; Rucker et al., 2012). On the contrary, an individual with high-power pays more attention to the utility of the product, and emphasis more on the quality and performance of the product rather than the symbolic meaning (Jin and Zhu, 2016). Meanwhile, the power affects consumer’s focus on goals and values. According to the Agentic-Communal Orientation Theory of power, people with high-power have agentic orientations, and they are more self-focused and this leads them to be less charitable (Han et al., 2017), willing to purchase products for themselves (Rucker et al., 2011). However, powerless individuals are more community orientated. Specifically, they pay more attention to others’ needs (Piff et al., 2010), and spend more for others (Rucker et al., 2011). Finally, the power experience has an effect on consumer’s behavioral tendency. According to the “Approach/Inhibition” Theory of power, an increase in power experience will activate individuals’ approach behavior and make them easier to perceive information such as rewards and success; while, a decrease in power experience will activate the inhibition behavior, and it is easier for individuals to perceive more information on threats and failures (Inesi, 2010).

It is worth noting that the possession of power is not accompanied only by the psychological experience. People often observe how the powerful and the powerless behave, they may come to hold a variety of expectations for the roles tied to different levels of power. The expectations of power are defined as the cognitive associations or schemas people have regarding how people behave based on their position of power (Rucker et al., 2014). The psychological experience of power refers to how one feels, and the expectations of power concentrate on how people with different power should behave in their actions. The expectations of power reflect organized knowledge structures and beliefs about how people should behave based on a role, can also guide consumer decision. For example, Rucker et al. (2014) found that powerful people would be more willing to choose status-related products which match their high-power experience when they are expected to be decent. People can behave in a manner consistent with the cognitive associations tied to a particular construct or role because those schemas become more accessible in one’s mind.

Despite the plethora of research that has examined how the psychological experience of power influences consumer behavior. For example, previous research has examined the impact of power on consumer’s goal pursuit (Chen J. et al., 2014), information processing (Smith and Trope, 2006; Chen J. E. et al., 2014), and consumption decision (Rucker et al., 2012; Garbinsky et al., 2014). However, to our best knowledge, the relationship between the expectations of power and impulsive buying remains relatively unexplored. we introduce the notion that power is accompanied by both an experience and expectations. As a consequence, for the same individual, focusing on the experience of power may produce a given set of effects on impulsive buying, whereas focusing on expectations of power may sometimes elicit a different desire to purchase.

### The experience of power and purchase impulsiveness

Lacking power is an aversive state and thus individuals are often motivated to reduce a state of powerlessness (Rucker and Galinsky, 2008). Consumers with low-power experience are eager to get rid of this negative psychological feeling in various ways, including compensating consumption (Chen et al., 2017). A focus on one’s internal psychological experience of power produces a focus on what a product will do for an individual (Rucker and Galinsky, 2009), as a result, hedonic products were predicted to be particularly valued by the powerless as a means of elevating a negative feeling. In addition, people with low power have less self-regulatory resources, while selection and self-regulation can consume the internal resources of individuals (Baumeister et al., 1998). Powerless are more dependent on emotions for making decision due to their limited cognitive processing resources (Shiv and Fedorikhin, 1999). When people rely on emotions to make decisions, it is easier for them to purchase hedonic products (Jin and Zhu, 2016). Both limited cognitive ability and negative experience cause people with low power more likely to buy hedonic products on impulse than utilitarian ones.

On the contrary, individuals with high-power experience will have a stronger sense of control because they could control more valuable resources (Berdahl and Martorana, 2006). Hence, they have less psychological demand for compensatory consumption. In terms of the choice of product features, people with a high sense of power pay less attention to the symbolism of the product, but more to the functional value of the product (Magee and Galinsky, 2008). High power leads to a preference for products that provide individuals with the greatest utility (Rucker and Galinsky, 2009). Prior study has confirmed that when focusing on the psychological experience of power, consumers with high power are more likely to have impulsive desire for utilitarian products (Jin and Zhu, 2016). Thus, we hypothesize that:

Hypothesis 1: Consumers with high-power experience have stronger impulsiveness to purchase utilitarian product than hedonic product.

Hypothesis 2: Consumers with low-power experience have stronger impulsiveness to purchase hedonic product than utilitarian product.

### The expectations of power and purchase impulsiveness

There are a number of reasons why the expectations of power may weaken powerless individuals’ desires for hedonic products. First, according to the “desire-willpower” theory of impulsive buying (Hoch and Loewenstein, 1991), When consumers have a time-inconsistent preference, they will experience the process of psychological conflict and struggle between purchase desire and willpower. Consumers will evaluate the reasonableness of their impulsive purchase of hedonic products (Rook and Fisher, 1995). In fact, consumers who have the desire to buy may not really make impulsive purchases, and there are no uncontrollable impulses in the world (Rook, 1987), Impulse buying is caused by the failure of self-control, and it is not a completely unthinking and uncontrollable behavior of consumers in essence (Baumeister, 2002).

Second, social stereotypes generally believe that people with low power should be more economical in their daily life because they have less valuable resources, and there is no need to consume unnecessary hedonistic products (Magee and Galinsky, 2008). When focusing on the expectations of power, the schemas and scripts about lacking power become more accessible in powerless peoples’ mind, they will not find a “reasonable” reason to buy hedonic products on impulse. Hedonistic products are mainly characterized by aesthetic and emotional experience, although they can bring immediate satisfaction to consumers (Pang et al., 2014), compared with utilitarian products characterized by instrumentality and functionality, it is difficult for consumers to prove the reasonableness of purchase (Dhar and Wertenbroch, 2012). Consumers will pay attention to hedonistic needs only after the necessary functional needs have been met, unless they can prove that they have the right to indulge (Chitturi et al., 2008). Powerless individuals have less quantity of valuable resources, and limited resources should be used to meet basic functional needs. When they are unable to prove the rationality of choosing pleasure goods, they are naturally unwilling to indulge themselves to consume hedonic products (Levav and McGraw, 2009).

In contrast, when high power individuals think about how powerful people should spend from the perspective of other people in society, they will find that social stereotypes generally believe that people with high power have more valuable resources, so they are more qualified and able to have fun, and should engage in consumer behavior consistent with their high power (Magee and Galinsky, 2008). The activation of schemas and scripts of powerful people leads to a spreading activation of constructs that can nudge high power people’s behavior in a manner consistent with those schemas. Thus, People with a high sense of power will think that it is very reasonable for them to consume hedonic products on impulse. Accordingly, we propose two specific hypotheses:

Hypothesis 3: When the expectations of power are activated, consumers with low-power experience will reduce impulsiveness to purchase hedonic product.

Hypothesis 4: When the expectations of power are activated, consumers with high-power experience will increase impulsiveness to purchase hedonic product.

### Deservingness

Deservingness inherently refers to a rationale for why someone is worthy of a particular outcome or treatment (Cavanaugh, 2014). As an important source of justification of consumption, deservingness often appears in advertisements. For example, firms often appeal to consumers’ sense of deservingness to encourage their consumption behavior with slogans such as “you deserve to have this good car” (GM), “today, you deserve to have a rest” (McDonald’s), and “you deserve it” (L’Oréal).

Research has proved that deservingness affects people’s indulgent consumption behavior (Xu and Schwarz, 2009). In the consumption research domain, impulsive behavior has been linked with “being bad” and with negative consequences in the areas of personal finance, post-purchase satisfaction, social reactions, and overall self-esteem (Rook and Fisher, 1995). Consumers who have purchase impulsiveness need to conduct a normative evaluation, i.e., judgments about the appropriateness of engaging in impulsive purchasing behavior (Rook and Fisher, 1995). Hedonic products can bring instant gratification compared with utilitarian products, but it is difficult to prove the normalization of hedonic consumption (Dhar and Wertenbroch, 2012). Compared with utilitarian purchase, consumers would provide more convincing proofs of justification for hedonic purchase (Khan and Dhar, 2010). On the contrary, utilitarian products which satisfy basic needs can naturally prove consumer’s justification (Kivetz and Zheng, 2017). Indulging with a reason refers to a rational or justified indulgence that feels like it is earned or deserved (Xu and Schwarz, 2009). For example, Kivetz and Simonson (2002) found that consumers are more likely to choose hedonic returns rather than practical returns if hedonic rewards require more efforts, because these efforts make them believe that they have the right for indulgence. Kivetz and Zheng (2006) found that people who work hard or exceed their tasks are more likely to choose hedonic products as rewards because they have logical reasons for their indulgent consumption. Kivetz and Zheng (2017) found that price promotion provides reasonable proofs for hedonic consumption compared with quantity discounts, and a consumer will think “I have the power to do so.” In contrast, Cavanaugh (2014) found that when people are reminded that they don’t possess a valuable relationship, deservingness would be reduced and thereby constrain their indulgent consumption.

**FIGURE 1:**
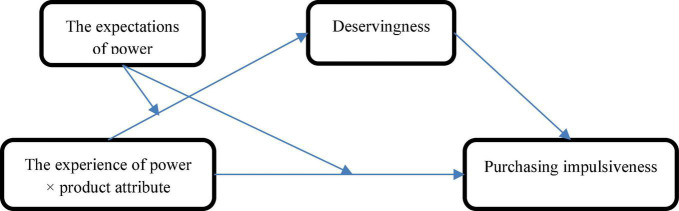
Conceptual diagrams of model.

On the basis of existing research, we propose that powerless people who make their impulsive purchase decisions through the observation of other’s behavior have a low sense of deservingness, and they cannot prove the justification of impulsively purchasing hedonic products. On the contrary, when power expectation is activated, the scripts and schemas about how powerful people consume goods increase their attention to hedonic products, and powerful people will feel that they are qualified and capable, that is, they are worth buying hedonistic products, thus providing a reasonable cause for hedonistic consumption. The conceptual diagrams of model are shown in Figure 1. Thus, we suggest the following:

Hypothesis 5: When power expectation is activated, consumers with low-power experience will have lower sense of deservingness, whereas consumers with high-power experience will have higher sense of deservingness.

Hypothesis 6: When power expectation is activated, deservingness plays a mediation role in the impact of power experience on impulsiveness to purchase utilitarian product versus hedonic product.

## Study design

### Study 1: The experience of power and purchase impulsiveness

The goal of study 1 was to test the H1 and H2. We manipulated subjects’ power experience and product attributes, and then asked them to response their impulsive purchase intention for utilitarian or hedonic products.

#### Pretest

First, consistent with prior work (Galinsky et al., 2003), we manipulated power experience consisting of an episodic recall task that asks subjects (under graduation students, *N* = 54) to recall an event in which either they have power over someone else (high power) or someone else have power over them (low power). The existing study has verified that this method has good reliability and validity (Rucker and Galinsky, 2008). After the manipulation, subjects were asked to report their power experience in a seven-point item “how powerful did you feel when completing the recall task?” (1 = not powerful at all, 7 = very powerful). At the same time, the subjects were asked to answer “to what extent you engaged in the episodic recall task?” (1 = not at all engaged, 7 = very much engaged). The results showed that all subjects could focus on power experience scenario (*M* = 6.13, *SD* = 0.65). Meanwhile, subjects in the high-power context reported their feelings were significantly more powerful (*M* = 5.26, *SD* = 1.26) than those in the low-power context (*M* = 3.07, *SD* = 0.96, *F*(1, 52) = 51.541, *p* < 0.001), indicating a successful manipulation of power experience.

Second, we chose portable music player as the stimulus material and manipulated its hedonic and utilitarian attributes (Pang et al., 2014). Here, the “hedonic attribute” refers to the aesthetic, experiential, and enjoyment-related benefits; and “utilitarian attribute” refers to the functional, instrumental, and practical benefits of consumption offerings (Chitturi et al., 2007). Subjects (under graduation students, *N* = 22) were asked to evaluate four important product attributes of a music player, including two hedonic attributes (appearance: changeable color; sound quality: high quality stereo audio) versus two utilitarian attributes (battery capacity: 20 h endurance; manipulative mode: control by earphone wire). They reported their feelings of each attribute with two seven-point items: “to what extent you think it is the hedonic (utilitarian) attribute of the music player” (1 = strongly disagree, 7 = strongly agree) (Chitturi et al., 2007). The results demonstrated that the battery capacity (*M*_hedonic_ = 3.09, *SD* = 0.75 vs. *M*_utilitarian_ = 6.41, *SD* = 0.67, *t* (21) = 16.461, *p* < 0.001) as well as manipulative mode (*M*_hedonic_ = 3.14, *SD* = 0.71 vs. *M*_utilitarian_ = 6.45, *SD* = 0.67, *t* (21) = 21.730, *p* < 0.001) were more utilitarian, and appearance (*M*_hedonic_ = 5.91, *SD* = 0.68 vs. *M*_utilitarian_ = 3.14, *SD* = 0.94, *t* (21) = 10.241, *p* < 0.001) as well as sound quality (*M*_hedonic_ = 5.95, *SD* = 0.72 vs. *M*_utilitarian_ = 5.27, *SD* = 0.87, *t* (21) = 12.396, *p* < 0.001) were more hedonic, indicating a successful manipulation of product attribute. Subjects also rated the importance of each attribute with a seven-point item: “to what extent you think this attribute is important for you to decide to buy this music player?” (1 = not important at all, 7 = very important). The results showed that there were no significant differences among four attributes (*F*(3, 84) = 0.548, *p* = 0.651).

#### Design and procedure

A total of 108 undergraduate students (*M*_age_ = 20.53, *SD* = 1.38, 56.82% females) in a university participated in the study for course credit. Subjects were randomly assigned to one of four conditions in a 2 (the experience of power: high vs. low) × 2 (product attribute: hedonic vs. utilitarian) between-subjects design. Firstly, we manipulated the experience of power with the same method used in the pretest study. After the manipulation of power, the subjects reported their mood with two seven-point items: “now, I feel sad (or happy)” (1 = strongly disagree, 7 = strongly agree). Subjects didn’t feel different positive affect [*M*_high–power_ = 4.19, *M*_low–power_ = 4.28, *F*(1, 106) = 0.27, *p* < 0.605, ns] or negative affect [*M*_high–power_ = 2.74 vs. *M*_low–power_ = 2.65, *F*(1, 106) = 0.29, *p* < 0.592, ns] in different power contexts, indicating that the experience of power had no effect on subjects’ mood. The results were consistent with the previous studies (Galinsky et al., 2003; Smith and Trope, 2006).

Then, we adopted the impulsive purchase scenario designed by Rook and Fisher (1995): Someone was going to buy a product, but occasionally met another ideal product. Under the condition of limited funds, how to make a choice in face of temptation could be viewed as the impulsive purchase. Subjects were exposed to the scenario:

A few days ago, you got a part-time job salary of ¥500 ($1 = ¥6.9) which you could control freely. Now, you need to buy a calculator necessary in your mathematics course. At the end of the week, you go to the shopping mall with the money and a credit card to buy the calculator (priced at about ¥100). But when you walk through the mall, you find a portable music player (priced at ¥399) is selling fantastically. You like it very much.

Meanwhile, the manipulations of two different product attributes in different designs were same to the pretest study.

After reading this scenario, in order to assess the purchase impulsiveness, subjects were instructed to select which one of five purchase decision alternatives they would make. These choice alternatives were designed to represent varying levels of purchase impulsiveness. From low to high impulsiveness, these alternatives were: (1) buying the calculator only, (2) wanting the portable music player but not buying it, (3) deciding not to buy the calculator, (4) buying both the calculator and the portable music player with the credit card, and (5) buying these plus a matching earphone with the credit card. This method was used and verified to effectively measure the purchase impulsiveness by many previous studies (e.g., Rook and Fisher, 1995; Luo, 2005). The impulsiveness of each purchase alternative was validated with an independent sample of students (under graduation students, *N* = 89). They were asked to rate the impulsive of each purchase alternative on a seven-point scale, the results showed that there are significant differences in the scores of impulsiveness of purchase between groups [*F*(4, 84) = 67.609, *p* < 0.001; *M_1_* = 1.41, *SD* = 0.51; *M_2_* = 2.29, *SD* = 0.69; *M_3_* = 3.30, *SD* = 0.73; *M_4_* = 3.94, *SD* = 0.90; *M_5_* = 5.11, *SD* = 0.76]. Counter to our expectation, not buying the calculator was viewed as more impulsive than either buying them only or wanting the portable music player. Because the script was described as planning to buy the calculator, some respondents appeared to view the change of plans as impulsive.

#### Results

##### Manipulation check

As expected, subjects in the high-power condition reported that their feelings were significantly more powerful (*M* = 5.20, *SD* = 1.04) than those in the low-power condition [*M* = 3.28, *SD* = 1.14, *F*(1, 106) = 84.53, *p* < 0.001], indicating a successful manipulation of power experience. Likewise, the results of manipulation check for product attribute priming were almost identical to the pretest study, indicating a successful manipulation of product attribute.

##### Purchase impulsiveness

**FIGURE 2:**
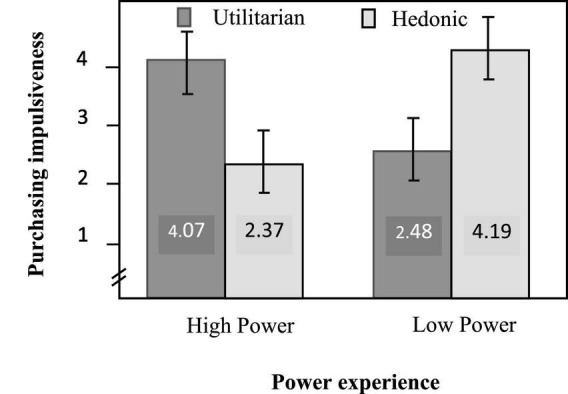
Results of study 1.

The results of ANOVA analysis showed that the main effect of the experience of power on purchase impulsiveness was not significant [*F*(1, 104) = 0.235, *p* < 0.629]. The main effect of product attribute on purchase impulsiveness was also not significant [*F*(1, 104) = 0.390, *p* < 0.534]. But the interactions between power and product attribute were significant [*F*(1, 104) = 91.617, *p* < 0.001]. Further results revealed that subjects’ impulsiveness to purchase utilitarian music player was significantly higher (*M* = 4.07, *SD* = 0.83) than hedonic music player [*M* = 2.37, *SD* = 0.69, *F*(1, 104) = 40.028, *p* < 0.001] in the high-power condition. In the low-power condition, subjects’ impulsiveness to purchase hedonic music player was significantly higher (*M* = 4.19, *SD* = 0.92) than utilitarian music player [*M* = 2.48, *SD* = 1.19, *F*(1, 104) = 51.978, *p* < 0.001]. The statistical diagram is shown in Figure 2.

#### Discussion

Study 1 shed light on the impact of the experience of power on impulsiveness to purchase products different attributes. The results demonstrated that subjects with high-power experience preferred utilitarian product more significantly compared with hedonic choice. On the contrary, subjects with low-power experience preferred the product with hedonic attribute rather than utilitarian product, which provided support for H1 and H2. Because powerful consumers have a stronger mentality of utility, individuals’ intentions and behavior are more consistent with their values when they have greater power (Magee and Smith, 2013), and thus they prefer products that could bring them functional value (Magee and Galinsky, 2008). Therefore, powerful consumers are more likely to be attracted by the utilitarian attribute, which leads to a higher impulsive purchase intention for these products. On the contrary, powerless consumers rely on emotions to make decisions (Jin and Zhu, 2016),they get immediate gratification by buying hedonic products.

### Study 2: Robustness of the impact of the experience of power on purchase impulsiveness

In study 1, we manipulated subjects’ power experience using the method of an episodic recall. In order to test the robustness of our findings in study 1, we conducted study 2 using an alternative measure of chronic power experience with a different stimulus material in a new context.

#### Pretest

In study 2, we chose a laptop as the stimulus material and manipulated it hedonic and utilitarian attributes according to the previous research (Chitturi et al., 2008). Subjects (under graduation students, *N* = 23) were randomly assigned to one of two groupings and read information about the different attributes of a laptop. The laptop was described as a combination of three utilitarian or three hedonic attributes respectively. The utilitarian dimension included the level of processing speed, memory size, and audio clarity. The hedonic dimension consisted of screen size, color, and weight. We combined these attribute descriptions with two pictures of the different laptops (see Supplementary Appendix Table 1). Then subjects evaluated each of three product attributes in different groupings with two seven-point items: “to what extent you think it is the utilitarian (hedonic) attribute of the laptop” (1 = strongly disagree, 7 = strongly agree). The attractiveness of the laptop was measured in the descriptions with a seven-point item (attractiveness: 1 = not attractive at all, 7 = very attractive). The results demonstrated that processing speed [*M*_hedonic_ = 2.29, *SD* = 1.23 vs. *M*_utilitarian_ = 5.75, *SD* = 1.22, *t* (11) = 16.14, *p* < 0.001], memory size [*M*_hedonic_ = 2.67, *SD* = 0.96 vs. *M*_utilitarian_ = 5.82, *SD* = 1.09, *t* (11) = 15.71, *p* < 0.001] as well as audio clarity [*M*_hedonic_ = 3.28, *SD* = 1.25 vs. *M*_utilitarian_ = 5.05, *SD* = 0.94, *t* (11) = 7.46, *p* < 0.001] were more utilitarian, and screen size [*M*_hedonic_ = 5.08, *SD* = 0.77 vs. *M*_utilitarian_ = 3.97, *SD* = 1.28, *t* (10) = 4.21, *p* < 0.05], color [*M*_hedonic_ = 6.21, *SD* = 1.21 vs. *M*_utilitarian_ = 2.38, *SD* = 1.19, *t* (10) = 25.17, *p* < 0.001] as well as weight [*M*_hedonic_ = 5.35, *SD* = 1.47 vs. *M*_utilitarian_ = 3.47, *SD* = 1.27, *t* (10) = 6.42, *p* < 0.01] were more hedonic, indicating a successful manipulation of product attribute. The results also showed that attractiveness had no difference between two groupings (*M*_hedonic_ = 4.01, *SD* = 1.52 vs. *M*_utilitarian_ = 4.27, *SD* = 1.87, *ns*).

#### Design and procedure

A total of 97 MBA students (*M*_age_ = 31.02, *SD* = 4.65, 60.82% females) in a university participated in the study for course credit. Subjects were randomly assigned to one of four conditions in a 2 (the experience of power: high vs. low) × 2 (product attribute: hedonic vs. utilitarian) between-subjects design. Different from study 1, subjects were asked to rate their agreement with eight items, e.g., “in my relationship with others, I think I have a great deal of power (1 = strongly disagree, 7 strongly agree),” to assess the power experience (see Supplementary Appendix A). As in previous research (Anderson and Galinsky, 2006), the scale showed high internal consistency (α = 0.96). Then, subjects were exposed to the scenario:

A few days ago, you got an annual bonus of ¥10,000. You want to buy a sport bicycle to take exercise at leisure time. At the end of this week, you go to the shopping mall with the money and a credit card to buy the bicycle (priced at ¥3,999) that you have followed with interests for some times. But when you walk through the mall, you find a new laptop (priced at ¥8,899) is selling fantastically. You like it very much.

Besides, descriptions for utilitarian or hedonic attributes of the laptop in different design groupings were same to the pretest study. And then the subjects were asked to answer: (1) buying the bicycle only, (2) wanting the laptop but not buying it, (3) deciding not to buy the bicycle, (4) buying both the bicycle and the laptop with the credit card, and (5) buying these plus a matching laptop bag with the credit card.

#### Results

##### Chronic power experience

We took the average scores of eight items of chronic level of power, ranking the average scores from high to low, and then carried out a median split. The results demonstrated that powerful subjects rated significantly higher scores (*M* = 5.27, *SD* = 0.78) than powerless ones [*M* = 2.77, *SD* = 0.61, *F*(1, 95) = 306.54, *p* < 0.001].

##### Manipulation check

Likewise, the results of manipulation check for product attribute priming were almost identical to that in pretest study.

##### Purchase impulsiveness

**FIGURE 3:**
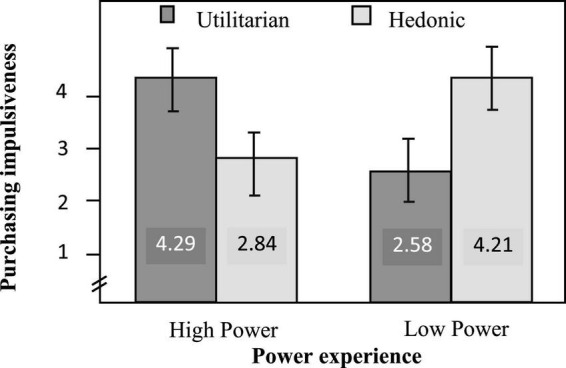
Results of study 2.

The ANOVA analysis showed that the main effect of chronic level of power on purchase impulsiveness was not significant [*F*(1, 93) = 2.075, *p* = 0.153]. The main effect of product attribute on purchase impulsiveness was also not significant [*F*(1, 93) = 0.017, *p* = 0.896]. But the interactions between power experience and product attribute were significant [*F*(1, 93) = 42.193, *p* < 0.001]. Specifically, subjects with high-power experience showed higher impulsiveness to purchase utilitarian laptop (*M* = 4.29, *SD* = 1.23) than hedonic laptop [*M* = 2.84, *SD* = 1.02, *F*(1, 93) = 15.193, *p* < 0.001]. Subjects with low-power experience had higher impulsiveness to purchase hedonic laptop (*M* = 4.21, *SD* = 1.18) than utilitarian laptop [*M* = 2.58, *SD* = 1.25, *F*(1, 93) = 27.171, *p* < 0.001]. See Figure 3 for detailed results.

#### Discussion

We replicated the findings in study 1 by measuring subject’s chronic level of power in a different consumption condition. As predicted, study 2 also provided the evidence that the impact of power experience on consumer impulsiveness to purchase products with utilitarian or hedonic attributes was robust. As expected, consumers with high chronic power experience were more likely to impulsively buy utilitarian products, whereas those with low-power experience were more likely to be fond of hedonic products. Taken together, studies 1 and 2 provided converging evidences for the hypothesized effect of power experience on relative preference for utilitarian versus hedonic products, and study 2 further verified the findings in the condition of chronic power experience. However, besides the power experience, consumers would also have power expectations. Different from the effect of power experience, power expectation perhaps has a completely different effect on impulsiveness to purchase utilitarian or hedonic products. In the next study, we demonstrated that whether power expectation would moderate the relationships between the power experience and impulsiveness to purchase utilitarian or hedonic products.

### Study 3: The moderate effect of the expectations of power

The purpose of study 3 was to test the moderating effect of the expectations of power on the impact of power experience on impulsiveness to purchase utilitarian or hedonic products.

#### Pretest

This pretest was conducted to examine the manipulation of the expectations of power according to the existing study (Rucker et al., 2014). Subjects (under graduation students, *N* = 54) were randomly assigned into one of two conditions, in which they were asked about their expectations for either high or low power regarding individual’s hedonic consumption. Subjects rated their agreement with the statements that individuals with high or low power would “own possessions associated with hedonic products” (1 = strongly disagree, 7 = strongly agree), “buy hedonic products” (1 = strongly disagree, 7 = strongly agree). We add up the scores of the two items (α = 0.89) and then average them, and the results of the ANOVA verified that subjects expected powerful people to be more likely to possess or buy hedonic products (*M* = 5.11, *SD* = 0.96) than the powerless ones [*M* = 2.91, *SD* = 0.71, *F*(1, 52) = 91.680, *p* < 0.001].

Next, in this study we chose trainers as the stimulus material and manipulated their utilitarian and hedonic attributes according to the previous research (Crowley et al., 1992). Subjects (under graduation students, *N* = 42) were randomly assigned to one of two groupings. The trainers were described as a combination of the two utilitarian aspects (wear resistance and protection) or two hedonic aspects (color and style). In the utilitarian grouping, the trainers were described as follows:

There is a pair of very practical trainers. The trainers are very durable so that they don’t fear wear and tear anywhere and anytime. At the same time, the sole has high-tech protective air cushion, which keeps your ankle away from hurt when you take exercises.

In the hedonic grouping, the trainers were described as follows:

There is a pair of very fashionable trainers with red, white, black, green, orange, and blue colors for you to choose. Besides, the trainers are also a “style king” so that you will feel free and casual, and they could highlight your charm and fashion when wearing them.

Then subjects evaluated each of two product attributes in different groupings with a seven-point item: “to what extent you think it is the utilitarian (hedonic) attribute of the trainers” (1 = utilitarian, 7 = hedonic). The attractiveness of the trainers was measured with a seven-point item (attractiveness: 1 = not attractive at all, 7 = very attractive). The results demonstrated that wear resistance [*M*_hedonic_ = 2.95, *SD* = 0.86 vs. *M*_utilitarian_ = 5.76, *SD* = 0.77, *F*(1, 40) = 123.879, *p* < 0.001] as well as protection [*M*_hedonic_ = 2.52, *SD* = 0.80 vs. *M*_utilitarian_ = 5.38, *SD* = 0.80, *F*(1, 40) = 121.622, *p* < 0.001] were more utilitarian, and color [*M*_hedonic_ = 5.24, *SD* = 0.62 vs. *M*_utilitarian_ = 2.67, *SD* = 0.73, *F*(1, 40) = 150.309, *p* < 0.001] as well as style [*M*_hedonic_ = 5.38, *SD* = 0.80 vs. *M*_utilitarian_ = 2.86, *SD* = 0.85, *F*(1, 40) = 97.197, *p* < 0.001] were more hedonic, indicating a successful manipulation of product attributes. The results also showed that attractiveness had no difference between two groupings [*M*_hedonic_ = 5.38, *SD* = 0.80 vs. *M*_utilitarian_ = 5.14, *SD* = 0.65, *F*(1, 40) = 1.106, *p* = 0.299].

#### Design and procedure

A total of 165 undergraduate students (*M*_age_ = 20.82, *SD* = 1.09, 61.21% females) in a university participated in the study for course credit. Subjects were randomly assigned to one of eight conditions in a 2 (the experience of power: high vs. low) × 2 (the expectations of power: yes vs. no) × 2 (product attribute: hedonic vs. utilitarian) between-subjects design.

Firstly, prior to presenting what happened in the scenario, subjects were first asked to “write down your relationship with the person who you had power over (who had power over you),” and then asked to describe “what happened during the event referring to that person and how you felt during the event referring to that person” (Rucker et al., 2014). Then, subjects completed the same manipulations in study 1 and were asked “how powerful did you feel when completing the recall task” (1 = not powerful at all, 7 = very powerful). They also were asked to rate “to what extent you engaged in the scenario role task?” (1 = not at all engaged, 7 = very much engaged). The mood was also measured, and results didn’t show different positive affect [*F*(1, 163) = 0.38, *p* < 0.539, *ns*] or negative affect [*F*(1, 163) = 1.071, *p* < 0.302, *ns*] in different power contexts.

Subsequently, we adopted the episodic priming method used in prior research (Rucker et al., 2014) to manipulate the expectations of power. Subjects were first asked to “write down the name or title of the role you held” and they were then asked to “describe what other people generally expect from someone in this role or similar roles and the stereotypes associated with this role.” In the condition with power expectation priming, we measured the subjects’ power experience and expectation by asking two questions: “how powerful did you feel when completing the recall task?” (1 = not powerful at all, 7 = very powerful) and “to what extent you anticipated the behavior of the role you held in the situation?” (1 = not at all anticipated, 7 = very much anticipated). The subjects in the condition without power expectation priming needed to do an irrelevant task, i.e., “imagine the place where you want to travel in the future.” Then, subjects were exposed to the scenario:

A few days ago, you just got a part-time job salary of ¥500 which you could control freely. Now, you need to buy a calculator needed in your mathematics course. At the end of this week, you go to the shopping mall with the money and a credit card to buy the calculator (priced at about ¥100). But when you walk through the shopping mall, you find a pair of trainers (priced at ¥489) is selling fantastically. You like them very much.

Besides, manipulations for utilitarian or hedonic attributes of the trainers were same to the pretest. And then the subjects were asked to answer: (1) buying the calculator only, (2) wanting the trainers but not buying it, (3) deciding not to buy the calculator, (4) buying both the calculator and the trainers with the credit card, and (5) buying these plus a matching T-shirt with the credit card.

#### Results

##### Manipulation check

As expected, subjects in the high-power condition reported that their feelings were significantly more powerful (*M* = 4.94, *SD* = 1.02) than those in the low-power condition [*M* = 3.14, *SD* = 1.21, *F*(1, 163) = 107.20, *p* < 0.001]. In addition, subjects reported a higher level of power experience involvement (*M* = 6.08, *SD* = 0.80), indicating a successful manipulation of power experience. The examination of power expectation showed that the subjects with high- or low-power experience had significant differences in the sense of power after the manipulation of power expectations [*F*(1, 82) = 116.983, *p* < 0.001]. When power expectation was activated, subjects in the high-power condition felt more powerful (*M* = 5.33, *SD* = 0.82) compared with those in the low-power condition (*M* = 3.29, *SD* = 0.90). The subjects reported a higher degree of power expectation involvement (*M* = 6.11, *SD* = 0.77). In addition, with regard to powerful people, there was no significant difference in the score of sense of power between the two groups that activated the expectations of power (*M* = 5.14, *SD* = 0.98) and did not activate the expectations of power [*M* = 4.74, *SD* = 1.04, *F*(1, 82) = 3.388, *p* = 0.069]; for people with a low sense of power, there was no significant difference in scores between the group with power expectation (*M* = 3.02, *SD* = 1.19) and the group without power expectation [*M* = 3.25, *SD* = 1.24, *F*(1, 79) = 0.699, *p* = 0.406]. Similarly, the results of manipulation check for product attribute priming were almost identical to the pretest study, indicating a successful manipulation of product attributes.

##### Purchase impulsiveness

The results of ANOVA analysis showed that the main effect of power experience on purchase impulsiveness was significant [*F*(1, 163) = 9.559, *p* < 0.01]. The main effect of product attribute on purchase impulsiveness was not significant [*F*(1, 163) = 2.250, *p* = 0.136]. The main effect of power expectation on purchase impulsiveness was also not significant [*F*(1, 163) = 0.436, *p* = 0.510]. The interactive effects of power expectation, power experience and product attribute on purchase impulsiveness were significant [*F*(1, 163) = 5.113, *p* < 0.05].

Specifically, after priming the power expectation, the main effect of power experience on purchase impulsiveness was significant [*F*(1, 79) = 53.773, *p* < 0.001]. The main effect of product attribute on purchase impulsiveness was not significant [*F*(1, 79) = 1.962, *p* = 0.165]. The interactions between power experience and product attribute on purchase impulsiveness were significant [*F*(1, 79) = 4.031, *p* < 0.05]. In terms of the subjects with high-power experience, there was a significant difference in purchase impulsiveness between utilitarian and hedonic trainers [*F*(1, 79) = 5.881, *p* < 0.05]; for the subjects with low-power experience, there was no significant difference in purchase impulsiveness between utilitarian and hedonic trainers [*F*(1, 79) = 0.182, *p* = 0.671].

When there was no power expectation priming, the main effect of power experience on purchase impulsiveness was not significant [*F*(1, 78) = 3.628, *p* = 0.061]. The main effect of product attribute on purchase impulsiveness was also not significant [*F*(1,78) = 1.905, *p* = 0.171]. The interactions between power experience and product attribute had a significant effect on purchase impulsiveness [*F*(1, 78) = 51.246, *p* < 0.001]. In terms of the subjects with high-power experience, there was a significant difference in purchase impulsiveness between utilitarian and hedonic trainers [*F*(1, 78) = 17.112, *p* < 0.001]; for the subjects with low-power experience, there was also a significant difference in purchase impulsiveness between utilitarian and hedonic trainers [*F*(1, 78) = 35.589, *p* < 0.001]. The results of the comparisons are shown in Table 1.

**TABLE 1:** Comparisons of purchase impulsiveness when the power expectation was activated or not.

Product attribute	High-power experience		Low-power experience	
	**Power expectation**		**Power expectation**	
	**Yes**	**No**	**Yes**	**No**
Utilitarian	4.48 (0.93)	4.62 (1.07)	3.29 (1.06)	3.45 (0.94)
Hedonic	5.24 (1.09)	3.33 (1.11)	3.15 (0.99)	5.35 (0.88)

*SDs* are presented in the parentheses.

#### Discussion

In study 3, we utilized an imagined role task to examine the impact of power experience on the impulsiveness to purchase utilitarian and hedonic products. More important, the study 3 extended the former two studies in the condition considering the moderate effect of power expectation. As presumed in H3 and H4, it was found that when power expectation was stimulated, consumers with low-power experience reduced impulsiveness to purchase hedonic products. Consumers with high-power experience increased impulsiveness to purchase hedonic products, but their impulsiveness to purchase utilitarian goods were not affected. In study 4, we extended our findings by verifying that when power expectation was activated, deservingness was an underlying mediation reason for the impact of power experience on the impulsiveness to purchase utilitarian or hedonic products.

### Study 4: The mediate effect of deservingness

The main objective of study 4 was to test H5 and H6. Specifically, when power expectation was activated, we expected that consumers with low-power experience would have lower perception of deservingness, whereas consumers with high-power experience would have higher perception of deservingness. Deservingness would play a role in mediating the impact of power experience on impulsiveness to purchase utilitarian or hedonic products.

#### Pretest

We manipulated situational power experience and power expectation through a task involving imagined roles. Subjects (under graduation students, *N* = 44) were told to imagine themselves either as a boss or an employee in a firm while reading a scenario describing that role (Rucker et al., 2014). Subjects in the high-power context read:

As a boss, you are responsible for directing your employees in making products. You have the right to decide the procedure of making products and the rules by which you appraise your subordinates. You evaluate your employees’ work performance quarterly but don’t give feedback of the final evaluation results to your employees. The employees have no rights to appraise your work.

In contrast, subjects in the low-power context read:

As an employee, you are in charging of making products according to your boss’s instructions. The boss determines the rules by which your work performance is to be appraised. As the employee, you must follow the orders of the boss. You will be appraised by the boss quarterly, and this evaluation results will not be given to you. You have no rights to appraise the boss.

After reading the scenario, subjects were asked to write about the role they were assigned, and answered “what the boss (employee) would think and how they would feel.” In addition, the subjects were asked to answer two questions: “how powerful did you feel with the role that you read?” (1 = not powerful at all, 7 = very powerful) and “to what extent you concentrated on the role that you read?” (1 = not at all concentrated, 7 = very much concentrated). Results demonstrated that subjects in the high-power condition showed significantly more powerful (*M* = 5.14, *SD* = 1.32) than those in the low-power condition [*M* = 2.91, *SD* = 0.97, *F*(1, 42) = 40.629, *p* < 0.001]. Meanwhile, subjects reported a higher involvement of power experience (*M* = 6.14, *SD* = 0.63).

In the power expectation priming task, subjects (*N* = 46) were asked to write about “what other people generally expect from someone in this role (boss or employee).” The power experience and the involvement degree of power expectation were measured in two seven-point items: “how powerful did you feel with the role that you read?” (1 = not powerful at all, 7 = very powerful) and “to what extent you anticipated the behavior of the role you held in the situation?” (1 = not at all anticipated, 7 = very much anticipated). The results showed that subjects in high-power expectation condition reported more powerful (*M* = 5.13, *SD* = 1.29) than those in low-power expectation condition [*M* = 2.78, *SD* = 0.95, *F*(1, 44) = 49.348, *p* < 0.001]. The subjects also reported a higher involvement degree of power expectation (*M* = 6.17, *SD* = 0.64).

In the pretest, we chose a smart wrist watch as the stimulus material and manipulated its utilitarian or hedonic attributes based on the previous research (Jin and Zhu, 2016). Subjects (under graduation students, *N* = 51) were randomly assigned to one of two groupings. In the utilitarian or hedonic groupings, subjects were exposed to a picture of a smart wrist watch with descriptions about its attributes in details respectively (see Supplementary Appendix Table 2). Then subjects evaluated the production attributes and attractiveness according to the descriptions with two seven-point items (attributes: 1 = hedonic, 7 = utilitarian; attractiveness: 1 = not attractive at all, 7 = very attractive). The results showed that the manipulation of product attributes was successful [*M*_hedonic_ = 3.19, *SD* = 1.13 vs. *M*_utilitarian_ = 4.64, *SD* = 1.29, *F*(1, 49) = 18.230, *p* < 0.001], and attractiveness had no difference between two groupings [*M*_hedonic_ = 5.27, *SD* = 0.72 vs. *M*_utilitarian_ = 5.08, *SD* = 0.70, *F*(1, 49) = 0.896, *p* = 0.348].

#### Design and procedure

A total of 252 undergraduate students (*M*_age_ = 20.71, *SD* = 1.10, 67.06% females) in a university participated in the study for course credit. Subjects were randomly assigned to one of eight conditions in a 2 (the experience of power: high vs. low) × 2 (the expectations of power: yes vs. no) × 2 (product attribute: hedonic vs. utilitarian) between-subjects design.

Firstly, we manipulated the experience of power with the same method used in the pretest study. Similarly, subjects also didn’t feel different positive affect [*F*(1, 250) = 0.275, *p* = 0.600, *ns*] or negative affect [*F*(1, 250) = 0.475, *p* = 0.491, *ns*] in different power contexts.

Subsequently, we adopted the same method used in the pretest study to manipulate power expectation. For people with a high sense of power, there was no significant difference in the score of their power when activating the expectations of power (*M* = 4.79, *SD* = 1.09) compared with not activating it [*M* = 5.00, *SD* = 1.06, *F*(1, 124) = 1.152, *p* = 0.285]; for people with a low sense of power, there was no significant difference in the score of their power when activating the expectations of power (*M* = 3.10, *SD* = 1.069) compared with not activating it [*M* = 3.21, *SD* = 1.08, *F*(1, 124) = 0.340, *p* = 0.561]. The subjects in the condition with power expectation priming reported a higher involvement degree of power expectation (*M* = 5.25, *SD* = 0.82). Subjects in the condition without power expectation priming were asked to do an irrelevant task, i.e., “imagine the place where you want to travel in the future.” Then, subjects were exposed to the scenario:

You need to buy a laptop used for your home job. On Sunday, you go to the shopping mall with ¥6000 and a credit card to buy a laptop (priced at ¥5589) that you have followed with interests for some times. But when you walk through the mall, you find a smart wrist watch (priced at ¥1899) is selling fantastically. You like it very much.

Besides, the manipulation of utilitarian and hedonic attributes of the watch in different design groupings was same to the pretest study. Purchase impulsiveness were measured through the questions: “would you likely buy this smart wrist watch?” (1- exactly not, 7- definitely yes).

Subsequently, to assess the deservingness, subjects rated with four seven-point items: “after reading the scenario, to what extent did you feel you deserve to” (1) “…reward yourself,” (2) “.treat.. yourself to nice things,” (3) “.indulge yourself a little,” and (4) “.buy something special for yourself.” (1 = “not at all deserving” and 7 = “extremely deserving”) (Cavanaugh, 2014). Items were combined into one deservingness measure (α = 0.96). To assess the purchase impulsiveness, we used the same method employed in study 1.

#### Results

##### Manipulation check

Subjects in the high-power experience condition reported feeling significantly more powerful (*M* = 4.90, *SD* = 1.08) than those in the low-power experience condition [*M* = 3.15, *SD* = 1.07, *F*(1, 250) = 166.841, *p* < 0.001], indicating a successful manipulation of power experience. Likewise, the results of manipulation check for product attribute priming were identical to the pretest, indicating a successful manipulation of product attributes. The reliability coefficient of deservingness was 0.91.

##### Purchase impulsiveness

The results of ANOVA analysis showed that the main effect of power experience on purchase impulsiveness was significant [*F*(1, 244) = 15.801, *p* < 0.001]. The main effect of product attribute on purchase impulsiveness was not significant [*F*(1, 244) = 0.337, *p* = 0.562]. The main effect of power expectation on purchase impulsiveness was not significant [*F*(1, 244) = 2.844, *p* = 0.093]. The interactive effects of power experience, power expectation and product attribute on purchase impulsiveness were significant [*F*(1, 244) = 36.098, *p* < 0.001].

When the subjects were activated to have the expectations of power, the main effect of power experience on purchase impulsiveness was significant [*F*(1, 122) = 54.186, *p* < 0.001]. The main effect of product attribute on purchase impulsiveness was not significant [*F*(1, 122) = 0.014, *p* = 0.907]. The interactions between power experience and product attribute had a significant effect on purchase impulsiveness [*F*(1, 122) = 4.541, *p* = 0.035]. In terms of subjects with high-power experience, there was not significant difference in purchase impulsiveness between utilitarian and hedonic watches [*F*(1, 122) = 2.526, *p* = 0.115], there was no significant difference in purchase impulsiveness between utilitarian and hedonic watches for subjects with low-power experience [*F*(1, 122) = 2.028, *p* = 0.157].

When the power expectation was not activated, the main effect of power experience on purchase impulsiveness was not significant [*F*(1, 122) = 1.281, *p* = 0.260]. The main effect of product attribute on purchase impulsiveness was also not significant [*F*(1, 122) = 0.445, *p* = 0.506]. The interactions between power experience and product attribute had a significant effect on purchase impulsiveness [*F*(1, 122) = 37.312, *p* < 0.001]. In terms of subjects with high-power experience, there was a significant difference in purchase impulsiveness between utilitarian and hedonic watches [*F*(1, 122) = 14.802, *p* < 0.001]. For subjects with low-power experience, there was also a significant difference in purchase impulsiveness between utilitarian and hedonic watches [*F*(1, 122) = 22.956, *p* < 0.001]. The results of the mean comparisons are listed in Table 2.

**TABLE 2:** Comparisons of purchase impulsiveness when the power expectation was activated or not.

Product attribute	High-power experience		Low-power experience	
	**Power expectation**		**Power expectation**	
	**Yes**	**No**	**Yes**	**No**
Utilitarian	4.63 (0.91)	4.84 (1.32)	3.68 (1.05)	3.81 (1.05)
Hedonic	5.03 (1.11)	3.71 (1.35)	3.31 (0.99)	5.22 (0.91)

*SDs* are presented in the parentheses.

The main effect of the impact of power experience on deservingness was significant [*F*(1, 244) = 26.629, *p* < 0.001]. The main effect of product attribute on deservingness was not significant [*F*(1, 244) = 0.539, *p* = 0.464]. The main effect of power expectation on deservingness was also significant [*F*(1, 244) = 5.794 *p* = 0.017]. The interactions between power experience, power expectation and product attribute had a significant impact on the deservingness [*F*(1, 244) = 38.550, *p* < 0.001]. The results of the comparisons of deservingness in different groupings are shown in Table 3.

**TABLE 3:** Results of comparisons of deservingness in different groupings.

Product attribute	High-power experience		Low-power experience	
	**Power expectation**		**Power expectation**	
	**Yes**	**No**	**Yes**	**No**
Utilitarian	4.45 (0.70)	4.84 (1.32)	3.46 (0.78)	3.77 (1.33)
Hedonic	4.90 (0.91)	3.70 (0.90)	3.15 (0.89)	4.93 (0.85)

*SDs* are presented in the parentheses.

##### Mediation analysis

**FIGURE 4:**
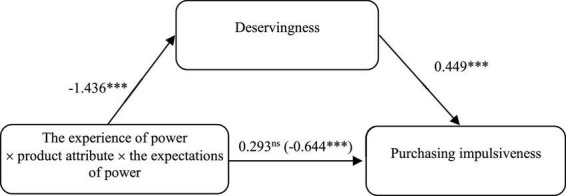
The mediate role of deservingness when the expectations of power were activated. ***p < 0.001.

To examine the mediate effect of deservingness, we conducted a bootstrapping analysis (model 8). With the power expectation as a moderator and deservingness as a mediator, we carried out the regression analysis (the sample size = 5000, CI = 95%) in which interactions between power experience and product attribute were an independent variable and purchase impulsiveness was used as a dependent variable. Conditional indirect effect results showed that the interaction effects of experience of power, product type and the expectations of power on deservingness is significant, β = −1.436, *SE* = 0.261, *CI* (−1.9492, −0.9220). Deservingness has a significant effect on purchase impulsiveness, β = −0.449, *SE* = 0.067, *CI* (0.3161, 0.5816), deservingness mediated the interactions between the experience of power, product attribute and the expectations of power on purchase impulsiveness, β = −0.644, *SE* = 0.167, *CI* (−1.0231, −0.3643). Hence, H5 and H6 were supported. The result of mediation test is shown in Figure 4.

#### Discussion

The convergent support for H3 and H4 in study 4 using different operationalizations of power and different measures of purchase impulsiveness is reassuring. Furthermore, we found evidence that the mechanism underlying this effect was a sense of deservingness. Specifically, deservingness mediated the effect of power experience on impulsiveness to purchase utilitarian and hedonic products when power expectation is activated. That is because consumers with low-power experience could not find the reasons to impulsively buy hedonic products when they image their consumption behavior through the schema and scripts of powerless people’s behavior. Social stereotypes generally posit that powerless people have less valuable resources and they should spent on necessary utilitarian products (Magee and Galinsky, 2008). Therefore, powerless people get a low sense of deservingness, and have no way to justify that they are qualified to consume hedonic products. Deservingness is a self-conscious emotion related to the evaluation of self-worth and capability, which can provide a reasonable reason for hedonic consumption (Cavanaugh, 2014). In contrast, social stereotypes generally believe that people with high-power are more qualified to have fun (Magee and Galinsky, 2008), the expectations of powerful people’s consumption to stimulate the hedonic motivation of people with high sense of power, and they can find the justification of “I deserve” for hedonistic impulsive buying.

## General discussion

A large number of studies have recently examined the impacts of power on various aspects of consumer behavior and decision-making (e.g., Rucker et al., 2012; Mourali and Yang, 2013; Duan et al., 2017; Li et al., 2022). This manuscript introduced a new theoretical perspective for understanding the effects of power in impulsive buying. Collectively, four studies support our theorizing regarding the interplay of the experience of power and the expectations of power on purchasing impulsiveness for different products. The results in studies 1 and 2 demonstrated that when focused on the psychological experience of power, consumers with either situational or chronic low-power experience would have higher impulsiveness to purchase hedonic products; whereas consumers with primed either situational or chronic high-power experience would have higher impulsiveness to purchase utilitarian products. However, study 3 showed that when focused on the expectations of power, powerless people would significantly reduce their impulsiveness to purchase hedonic products, and in terms of consumers with high-power, their impulsiveness to purchase hedonic products would increase significantly. Further, study 4 uncovered the mechanism underlying this phenomenon is that the expectations of power will induce consumers to think about how people with different power should do in consumption (Rucker et al., 2014), which in turn will lead consumers to evaluate the normalization of their consumption behavior (Rook and Fisher, 1995). The powerless consumers cannot justify the normative causes to purchase hedonic products because they don’t think they deserve them. On the contrary, powerful consumers will amplify the sense of “deservingness,” which leads to a significant increase in their impulsiveness to purchase hedonic products. Therefore, our findings verified that deservingness mediated the impact of power experience on impulsiveness to purchase products with different attributes when power expectation was activated.

### Theoretical contributions

We believe this research makes several contributions. Firstly, for the literature on impulsive buying, this work demonstrates a relatively novel effect: the moderating impact of the expectations of power on purchase impulsiveness. Previous studies have mainly focused on the impact of the experience of power on impulse purchase, and have not yet reached a consistent conclusion. For example, the power-approach theory has found that power activates a general tendency to approach whereas powerlessness activates a general tendency to inhibit. As a result, power was associated with an increased tendency to impulsive buying (Keltner et al., 2003). On the contrary, research based on the “construct level theory” has found that powerful people tend to make decisions at a high level of construction, and pay more attention to the long-term results of decisions, which enable them to avoid short-sighted behavior and are not easy to impulsively purchase products; while powerless people will show an adverse inclination (Smith and Trope, 2006). Hence, powerful consumers are more likely to be impulsive in consumption, while powerless ones are less likely to be impulsive. However, power is accompanied by both an experience and expectations, the link between power and behavior can critically depend upon whether an individual focuses on the experience or expectations of power (Rucker et al., 2014). Given these conflicting effects of power and impulsive buying, we first delineate a two-facet portrait of power in the role of affecting purchase impulsiveness by proposing a theoretical extension from the experience of power to the expectations of power. When focused on the experience of power, people concern about how an experience of power makes them feel and how they should respond based on those feelings (Rucker et al., 2014), thus, powerless individuals are more likely to buy hedonic products on impulse to get a positive experience of immediate gratification. In contrast, powerful individuals pay less attention to the symbolism of the product, but more to the functional value of the product (Magee and Galinsky, 2008), they are more likely to have impulsive desire for utilitarian products (Jin and Zhu, 2016). While the activation of schemas or scripts related to power produce a very differential outcome as is produced were one to focus on the internal experience of power. People activating the expectations of power focus on the cause of the impulsive buying, powerless people could not find out the cause to purchase hedonic products, because when focused on the actual cause of their impulse, they found themselves unable to indulge hedonic products. On the other hand, powerful people have the ability to engage in the consumption of hedonic products.

Secondly, the study further shed lights on the effect of consumers’ self-awareness emotions by identifying the mediation role of deservingness in the impact of three-way interactions between the experience of power, product attribute and the expectations of power on purchase impulsiveness. In this context, powerless consumers would experience lower sense of deservingness and could not prove their qualification and capability of “worth” indulging in consumption, because social stereotypes argue that powerless people have less valuable resources and thus should be simple and frugal (Magee and Galinsky, 2008). On the contrary, powerful people have more valuable resources, more ability and enjoyment (Rucker et al., 2014). In this scene, powerful consumers would spontaneously generate higher sense of deservingness, and produce a higher impulsiveness to purchase hedonic products. Deservingness is often discussed in marketing advertising, which has been proved to affect consumers’ purchase intentions. Although extant research has examined deservingness playing an important role in consumer decision-making (Cavanaugh, 2014), few research has systematically explored the role of deservingness on impulsive purchase behavior. Our research confirmed that consumers’ focus on either the psychological experience of power or the expectations of power can influence normative evaluations of impulsiveness to purchase utilitarian or hedonic products through the sense of deservingness. The conclusions would enrich and supplement the literature on the relationships between deservingness and impulsive purchase behavior.

A final contribution of this research is that it offers not only a new theoretical lens of power on purchasing impulsiveness, but suggests a tool of self-regulation. Prior studies show that highly impulsive buyers do not give in to every spontaneous buying demand, they will make the judgments about the appropriateness of making an impulsive purchase (Rook and Fisher, 1995), in fact, consumers’ impulse buying behavior is the result of the failure of self-control (Baumeister, 2002). We tested that one likely intervening factor arises from the expectations of power. When the expectations of power are activated, it is likely to elicit the normative evaluations about the appropriateness of making an impulsive purchase of hedonic products. In other words, the expectation of power act as a self-control mechanism for impulse purchase.

### Managerial implications

The findings also raise some practical suggestions for firms that want to stimulate consumer impulsive purchase behavior. First, marketers need to design marketing plan accurately according to product attributes and target consumers’ characteristics of power. Specifically, powerless consumers are more likely to buy hedonic products impulsively; whereas powerful consumers are more likely to impulsively purchase utilitarian products. Meanwhile, besides the chronic characteristics, the power perception may also be influenced by situational factors and consumers perhaps have the temporary power perception. For example, a boss who has an authority in a firm may also experience a temporary low power because of the unsuccessful contract negotiations. These findings suggest that in addition to observing the consumers’ chronic characteristics of power, a variety of measures such as background music, placement of products or sales person’s language can be taken to stimulate the consumer’s temporary power perception (Luo et al., 2016; Huang et al., 2017).

Moreover, firms need to conscientiously manage consumers’ power expectation. The power expectation refers to the social stereotypes that how and what consumers with different power should do in the opinions of other individuals, which can lead to consumers’ evaluation of consumption rationality. Hence, firms should take various ways to activate the power expectation for consumers with high power perception. Contrarily, firms should try to avoid the power expectation for consumers with low power perception, and guide them to focus on the positive experience of consumption or shorten their decision time.

Finally, marketers need to realize that the sense of deservingness is actually a double-edged sword. For powerful consumers, reminding of deservingness may enhance their impulsive purchase intention. But in terms of powerless consumers, deservingness perhaps reduces consumers’ impulsive purchase intention because they cannot find reasons for rationalization of consumption.

## Limitations and future research

This research also has some limitations that should be discussed in future research. Firstly, we demonstrated that power experience and power expectation can affect consumers’ impulsiveness to purchase utilitarian and hedonic products. The present research also confirmed that whether or not consumers having the sense of power expectation were one of the key influential factors of purchase impulsiveness. However, the question that what factors will be the antecedents of power expectation has not been discussed. For example, Rucker et al. (2014) assumed that interdependent self-construal individuals might be prone to consider the power expectation, and independent self-construal individuals might be more likely to focus on the power experience as they concentrate more on the self in comparison. Therefore, it is necessary to study the boundary conditions and moderators of the relationships between power expectation and purchase impulsiveness in future research.

Secondly, we verified that when the expectations of power were primed, the sense of deservingness would play a mediation role in the impact of interactions between power experience and product attribute on purchase impulsiveness. Deservingness is related to consumers’ positive psychological emotions (Xu and Schwarz, 2009). However, the impact of the expectations of power on consumers’ negative emotions, such as guilt, has not yet been studied. Guilt is a self-conscious emotion with negative valence, which can influence consumers’ self-control (Giroux et al., 2022). In future research, it is valuable to continue exploring the impact of the expectations of power on purchase impulsiveness with guilt as a mediator.

Finally, with the measurement method of deservingness, it was found that when the expectations of power were activated, powerless consumers had lower sense of deservingness, which in return would reduce consumers’ impulsiveness to buy hedonic products. In future research, we will manipulate powerless people’s sense of deservingness in order to observe their impulsiveness to purchase the hedonic products. We speculate that, when increasing the deservingness, even if power expectation were primed, powerless people would not reduce their impulsiveness to purchase the hedonic products inasmuch as they could enjoy their feeling of deservingness.

## Data availability statement

The original contributions presented in this study are included in the article/Supplementary material, further inquiries can be directed to the corresponding author.

## Ethics statement

Ethical review and approval was not required for the study on human participants in accordance with the local legislation and institutional requirements. Written informed consent for participation was not required for this study in accordance with the national legislation and the institutional requirements.

## Author contributions

All authors listed have made a substantial, direct, and intellectual contribution to the work, and approved it for publication.
