# Supplementary material for: From experience to expectation: The reverse effect of power on purchasing impulsiveness
Source: Front Psychol. 2023 Mar 8;14:1094536. doi: 10.3389/fpsyg.2023.1094536 (PMC10032043; doi:10.3389/fpsyg.2023.1094536)
Supplement: Supplementary file 1 [file Data_Sheet_1.pdf]

Appendix A. Supplementary Information

TABLE A1 Stimulus materials in study 2.

**Hedonic attribute**	**Screen size**	**Color**	**Weight**
	● Wide	● Many colors	● 0.6 kilograms
**Utilitarian attribute**	**Processing speed**	**Memory size**	**Audio clarity**
	● 8 GHz	● 120 GB	● High clarity

Note: The images will be available upon request, readers can contact the corresponding author to access these images.

Chronic level of power scale (1 = strongly disagree, 7 = strongly agree),

In my relationships with others,

______I can get people to listen to what I say.

______My wishes do not carry much weight.

______I can get others to do what I want.

______Even if I voice them, my views have little sway.

______I think I have a great deal of power.

______My ideas and opinions are often ignored.

______Even when I try, I am not able to get my way.

______If I want to, I get to make the decisions.

TABLE A2 Stimulus materials in study 4.

	Processor	Storage capacity	Camera	Hedonic functions	Color
**Hedonic attribute**	●800 MHz	●2 GB	●1.9 million pixel photo<br>●720P video	●Loudspeaker<br>●Microphone<br>●Sound photo	●Cool black<br>●Honorable white<br>●Space grey<br>●Boarse orangey<br>●Kinetic green<br>●Rose gold

	Processor	Storage capacity	Communication functions	Other functions	Color
**Utilitarian attribute**	●800 MHz	●8 GB	●Hands-free hearing<br>●Short message reminding<br>●Email reminding	●Smart Relay<br>●Voice control<br>●Memographer<br>●Voice Memo<br>●Emergency rescue	●Black

Note: The images will be available upon request, readers can contact the corresponding author to access these images.
